# Association of adipose tissue infiltration with cardiac function: scoping review

**DOI:** 10.1080/21623945.2025.2489467

**Published:** 2025-04-10

**Authors:** Mansour M. Alotaibi, Naif Z. Alrashdi, Marzouq K. Almutairi, Mohammed M. Alqahtani, Anwar B. Almutairi, Sami M. Alqahtani, Hamoud M. Alotaibi, Amani K. Bajunayd

**Affiliations:** a Department of Rehabilitation, Faculty of Applied Medical Sciences, Northern Border University, Arar, Saudi Arabia; b Center for Health Research, Northern Border University, Arar, Saudi Arabia; c Department of Physical Therapy and Health Rehabilitation, College of Applied Medical Sciences, Majmaah University, AL-Majmaah, Saudi Arabia; d Health and Basic Sciences Research Center, Majmaah University, Majmaah, Saudi Arabia; e Department of Physical Therapy, College of Applied Medical Sciences, Qassim University, Buraidah, Saudi Arabia; f Department of Respiratory Therapy, College of Applied Medical Sciences, King Saud bin Abdulaziz University for Health Sciences, Riyadh, Saudi Arabia; g King Abdullah International Medical Research Center, Riyadh, Saudi Arabia; h Department of Physical Therapy, Faculty of Allied Health, Kuwait University, Jabriya, Kuwait; i Ministry of Health, Aseer Health Cluster, Abha, Saudi Arabia; j Department of Community Health Sciences, College of Applied Medical Sciences, King Saud University, Riyadh, Saudi Arabia; k Department of Behavioral and Community Health, School of Public Health, University of Maryland, College Park, MD, USA; l Department of Internal Medicine, Western University, Ontario, Canada

**Keywords:** Adipose tissue infiltration, cardiac function, skeletal muscles, heart failure, biomarker

## Abstract

Evidence suggests that adipose tissue (AT) infiltration in skeletal muscles may negatively influence cardiac function, yet its use as a biomarker remains unclear. This scoping review examined the relationship between AT infiltration and cardiac function in adults. A systematic search of PubMed, CINAHL and SCOPUS identified peer-reviewed studies reporting AT infiltration and cardiac function measures. Excluded were review-type studies, animal studies, abstracts and case series. Study quality was assessed using the Study Quality Assessment Tool (SQAT). Three good-quality studies were included. Findings demonstrated a negative association between AT infiltration and cardiac function parameters, including exercise capacity, left ventricular ejection fraction (LVEF) and heart failure events, in cancer survivors and healthy individuals. There is evidence supporting an association between increased AT infiltration of skeletal muscles and impaired cardiac function, highlighting the need for further research to validate AT infiltration as a potential biomarker. Despite the limited available studies, our findings highlight a distinct association between skeletal muscle AT infiltration and cardiac dysfunction, independent of general obesity.

## Introduction

Cardiovascular disease (CVD) is the leading cause of mortality and disability across the world [1]. In 2019, it has been reported that approximately one-third of global deaths were attributed to CVD, with an estimated 17.9 million death [2]. Several demographic and other risk factors, including age, obesity (increased body fat) and sarcopenia, were found to aggravate the risk of mortality [1,3]. Further. early screening and prevention of cardiac impairments and reduced cardiovascular performance may reduce the rate of CVD-related deaths [3]. Other body composition factors were also found to impact cardiac function. Specifically, the reduced quality of the skeletal muscle, commonly known as sarcopenia, poses an additional risk on cardiac function [3], thereby increasing the risk of death. With ageing, several factors may contribute to deteriorating the quality of skeletal muscles and cardiac performance [3], including lack of physical activity, mitochondrial dysfunction, reduction in blood capillaries that supply muscles, insulin resistance and hormonal changes. These factors increase the likelihood of adipose tissue (AT) infiltration in skeletal muscles [4]. Yet, little evidence delineates the relationship between AT infiltration of skeletal muscles and cardiac performance. AT infiltration of skeletal muscles could serve as an important biomarker for cardiac function to guide clinician towards the prevention of this problem.

AT infiltration of skeletal muscle can present as inter-muscular adipose tissue (IMAT) or intra-muscular adipose tissue (IntraMAT) [5]. The clear distinction between these two types of fatty infiltration is defined by the location of the AT [5]. IMAT is the AT located between adjacent muscle groups and underneath the deep facia, whereas the IntraMAT is the AT infiltrated within or between muscle fibres [5]. IntraMAT is of particular interest due to its important role in affecting insulin sensitivity and contributing to metabolic syndrome [6–8], thereby increasing the likelihood of developing sarcopenia [9]. In turn, sarcopenia is a prognostic predictor for developing several heart diseases, including heart failure [9]. In addition, evidence suggests that increased IntraMAT is associated with reduced exercise capacity, as measured by whole-body peak oxygen consumption (VO_2_), particularly in breast cancer survivors who are known to be at risks to cardiac dysfunction [10]. The Framingham study found that the risks associated with IntraMAT extend to affecting physical functioning, such as walking speed and grip strength, in adults [11]. The same study showed that IntraMAT was more sensitive to impairments of physical function than visceral and subcutaneous adipose tissue [7,8,11], which may contribute to reducing cardiac function through reducing maximum cardiac capacity.

Cardiovascular magnetic resonance (CMR) imaging is considered as the gold standard measure of cardiac function during both rest and exercise, yielding reliable and reproducible outcomes [12,13]. This technique is superior to the traditional cardiac imaging at rest where the heart may compensate for undetectable dysfunction that is sensitive to maximum cardiac function, commonly observed with exercise [13]. Similarly, magnetic resonance imaging (MRI) is considered as the gold standard for IMAT and IntraMAT quantification, providing a reliable measure of muscle/fat fractions [5,14]. Several assessment tools of cardiac structure and function [15], IMAT infiltration [16] and IntraMAT infiltration [17] exist, such as echocardiography and computed tomography (CT). Nevertheless, clinicians often use body mass index (BMI) to articulate cardiac dysfunction to adiposity, which may lack accuracy compared to the outcomes of AT infiltration [18].

Examining the relationship between cardiac function and AT infiltration of skeletal muscles is important, specifically because often conditions, such as cancer, may lead to cachexia and increase AT infiltration in skeletal muscles. This consequently may impact overall cardiac function, thereby posing risks to the health status of affected individuals [19]. Given the breadth of research on this topic, this scoping review aimed to examine the extent and depth of the literature concerning the relationship between cardiac function and AT infiltration of skeletal muscles in adults.

## Materials and methods

### Identifying the research question

In this scoping review, we comprehensively and broadly searched the literature to generate breadth of coverage to synthesize the currently available evidence regarding the potential relationship between AT infiltration of skeletal muscles and cardiac function in adults. Thus, we asked the following question: *What is known in the literature concerning the relationship between cardiac function and AT infiltration of skeletal muscles in adults?* We did not aim to evaluate effect sizes or infer causality. Therefore, potential studies were limited to peer-reviewed human research with direct measures of AT infiltration and cardiac function. We reported our findings following the previously published framework for performing scoping reviews [20]. In this scoping review, we followed the Arkesy and O’Mally’s guidelines and recommendations for performing scoping reviews [20,21].

### Identifying relevant studies and study selection

We adopted our key terms and search strategy to locate relevant studies (Table 1) via three different databases, including PubMed, CINAHL and SCOPUS, searching from inception. We used the MeSH (medical subjects headings) technique for developing our search strategy, and we ran that search strategy in PubMed, and then we modified the search strategy accordingly for the other databases, to fulfil other databases’ technical requirements. Detailed information on the search was provided in Supplement 1. We included studies if they reported at least one measure of AT infiltration, one cardiac function measures and its full text was available in English language. We excluded review-type studies, animal studies, conference proceedings/abstracts or case studies. For the inclusion/exclusion process, two teams of reviewers independently reviewed titles and abstracts of all retrieved studies followed by solving any discrepancy in the inclusion decision by discussion and agreement. If an agreement could not be reached, a third reviewer (SA) was consulted and made the final decision. Next, the same two team of reviewers reviewed full texts for the inclusion decision, and discrepancy in the inclusion/exclusion decision was resolved by the same procedure illustrated above.

**Table 1.** Summary table participants and study characteristics for reviewed articles on the association of adipose tissue infiltration and cardiac function.

Author (year)	Design	Sample size	Age (years)	Cardiac Measure	AT Infiltration Measure	Main Findings	Relationship	Evidence Quality
Kirkham et al. [24]	Cross-sectional	16 Breast cancer survivors (BCS), 16 matched controls (MC) and 12 young controls (YC)	BCS:65 ± 10 MC:65 ± 10 YC: 25 ± 4	LV end-diastolic and end-systolic volumes, ejection fraction	IMAT was measured using MRI. Thigh fat fraction calculated as: IMF/(IMF + muscle) *100%.	increased AT infiltration of the thigh is associated with impaired peak VO2 in cancer survivors and controls.	-	Good
Huynh et al. [25]	Longitudinal	3075 population-based cohort, 799 tertile 1, low intramuscular fat; 800 tertile 2; 799 tertile 3, high intramuscular fat	LIT: 73.3 ± 2.8 T2: 73.5 ± 2.9 HIT: 73.9 ± 2.9	Incidence of heart failure	Mid-tight CT scan, lower attenuation coefficient (measured in Houns-field units [HU]) of the thigh obtained by CT scan was interpreted as greater intramuscular fat infiltration	After adjustment for age, sex, race, education, blood pressure, fasting blood sugar, current smoking, prevalent coronary artery disease and creatinine, higher intramuscular fat infiltration was associated with a higher risk of HF (HR: 1.34 [95% CI: 1.06–1.69]; *p* = 0.012, tertile 3 vs tertile 1). This association remained significant when intramuscular fat infiltration was analysed as a continuous variable (HR: 1.17 [95% CI: 1.06–1.29]; *p* = 0.002, per SD in HU)	-	Good
Reding et al. [23]	Cross-sectional	28 (14 cancer survivors > 12-months post-cancer treatment, and 14 controls)	54 ± 17	VO2 peak and exercise- associated measures of left ventricular ejection fraction (LVEF)	Ratio of IMF to skeletal muscle in the paraspinal muscles measured by MRI	Among cancer survivors that previously received anthracyclines and controls, increased intermuscular fat was associated with reduced VO2 peak even after accounting for exercise-associated cardiac changes. Increased IMAT was correlated with reduced resting and exercise associated with LVEF.	-	Good

'-' indicates inverse relationship.

### Charting the data

Our variables of interests include AT filtration and cardiac function in healthy adults. All review teams met and discussed key items of data that needed to be extracted from our included studies. Next, two authors (MMA; NZA) independently extracted relevant data, and then they met to discuss their data extraction sheets and resolved any discrepancy and reached a final data extraction sheet. Data extraction included year of publication, author name, study design, sample size, sample age, cardiac measure, AT infiltration measure, main findings, observed relationship (i.e. positive or negative) and evidence quality score. Finally, we performed a thematic analysis to produce a thorough summary of the information reported in the included studies.

### Study quality assessment

Commonly, study quality assessment is not required in scoping reviews [21]. However, we sought to evaluate the study quality for the included studies to further deepen our understanding regarding the currently available literature about the topic being studied. Thus, we chose the Study Quality Assessment Tool (SQAT) that was developed by the National Heart, Lung, and Blood Institute in 2013 [22]. This tool contains 14-specific question to evaluate internal validity, and produces and uses text-type decisions to rate studies and categorize them into poor, fair or good [22]. SQAT requires commenting on major methodological aspects of any included study that achieves a poor score [22].

## Results

### Search results

**Figure 1.**
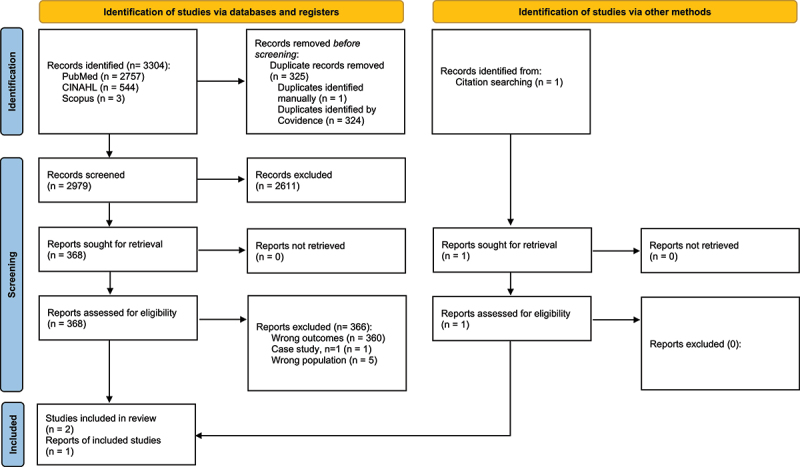
PRISMA flowchart for the results of the search.

Figure 1 depicts the summary of the article search. In brief, the search process yielded 3304 articles. After duplicates were removed (*n* = 325), the reviewers screened 2979 titles and abstracts and excluded 2611 articles based on the inclusion and exclusion criteria listed above, leaving 368 articles eligible for full-text screening. The reviewers excluded 370 articles that had the wrong outcomes (*n* = 360), wrong population (*n* = 5) and wrong study design (i.e. case study (*n* = 1)). Only two articles [23,24] remained after excluding these articles. In addition, the hand search resulted in the inclusion of an additional article [25]. Finally, this review yielded three articles [23–25].

### Characteristics of included studies

Of the three included studies [23–25], one study was conducted in Canada [24], and two studies were conducted in the United States [23,25]. All the included studies used observational study designs: Two studies used cross-sectional study design [23,24] and one study used longitudinal study design [25]. The reviewed studies recruited a total of 3,161 adults aged 25–70 y. Two studies recruited cancer survivors and controls [23,24] and one study recruited healthy adults only [24]. Finally, the SQAT assessment of evidence quality showed that all the included studies achieved good evidence quality based on aspects of internal validity.

### Outcomes of interest

A study by Reding et al. [23], used MRI to measure IMAT of the paraspinal muscles and used VO2 peak and left ventricular ejection fraction (LVEF) to assess cardiac function. Increased IMAT was correlated with reduced VO2 peak, resting LVEF and exercise associated LVEF in cancer survivors and control (*r* > .05) [23]. The study by Kirkham et al. [24], used MRI to assess IMAT of the mid-thigh and to evaluate cardiac function through measurements of left ventricle end-diastolic and end-systolic volumes and LVEF. Increased thigh IMAT was associated with reduced peak VO2 in cancer survivors and controls (*R*^*2*^~60%). The study by Huynh et al. [25] measured IntraMAT using CT scans and cardiac function as events of heart failure. After adjustment for age, sex, race, education, blood pressure, fasting blood sugar, current smoking status, prevalent coronary artery disease and creatinine level, increased IntraMAT was associated with a higher risk of HF (HR: 1.34 [95% CI: 1.06–1.69]; *p* = 0.012, tertile 3 vs tertile 1). This association remained significant when IntraMAT was analysed as a continuous variable (HR: 1.17 [95% CI: 1.06–1.29]; *p* = 0.002, per SD in HU).

## Discussion

The current scoping review answered the question of ‘What is known in the literature concerning the relationship between cardiac function and AT infiltration of skeletal muscles in adults?’ Three studies that met our selection criteria were included [23–25] and provided evidence of positive association of exercise capacity, LVEF and heart failure events, as measures of cardiac function, with AT infiltration of skeletal muscles in cancer survivors and healthy individuals. Two studies used cross-sectional study design [23,24] and one study [25] used longitudinal study design. One study [23] reported large and significant correlations between both resting LVEF and exercise-associated LVEF with IMAT of the paraspinal muscles in adult cancer survivors and controls. The second study [24] documented that IntraMAT of the thigh muscles significantly predicted VO2Max as a proxy for cardiac measure in adult cancer survivors and controls. The third study [25] reported a significant association between IntraMAT and heart failure events, after adjusting for demographic characteristics.

This scoping review found that limited evidence exists concerning the relationship between AT infiltration and cardiac measure, yielding an important unexplored research area. The currently available evidence on this relationship exhibits good evidence quality by addressing several aspects of internal validity, such as clearly defining the independent and dependent variables and adjusting for key potential confounders. However, there are no rigorous research designs, such as RCT, to determine if suppressing AT infiltration of skeletal muscles would improve cardiac function. This is important for two reasons: measurement of AT infiltration could provide a clinical value as a biomarker for cardiac function [26] and increased AT infiltration can be prevented by resistance exercise [27,28]. Nevertheless, due to the limited evidence, further studies are needed to confirm the association between AT infiltration and cardiac function.

It is important to distinguish between adiposity types when evaluating cardiac risk. General measures of obesity, such as BMI or waist circumference, do not capture ectopic fat distribution and may misclassify individuals with normal weight but high AT infiltration in skeletal muscle [18,29,30]. IMAT and IntraMAT, by contrast, are specific to the skeletal muscle compartment and reflect qualitative changes in muscle composition [5,31]. These fat depots can influence systemic metabolism, insulin resistance, and muscle contractility [6,29,30], which may indirectly impact cardiac function by reducing exercise tolerance and increasing circulatory stress [10,23,24]. Unlike intra- or pericardiac adipose tissue, which exerts local detrimental effects on the myocardium [32,33], IMAT/IntraMAT may represent a global metabolic and musculoskeletal phenotype associated with cardiovascular burden [4,9,34]. Furthermore, the adverse effects of epicardial and visceral adiposity on cardiac health are well-documented [32,33]. In contrast, skeletal muscle AT infiltration represents a distinct and underexplored ectopic fat depot. Unlike epicardial fat, which acts locally on the myocardium, IMAT and IntraMAT may contribute to cardiovascular dysfunction through systemic metabolic pathways, reduced physical function and impaired cardiorespiratory fitness [8,26,35].

The relationship between increased fat deposition in different areas of the body and impaired cardiac function is well documented in the literature. Specifically, evidence shows that increased epicardial fat volume, visceral fat and overall adiposity are associated with impaired cardiac function [32,33,36–39], establishing evidence of a detrimental effect of obesity on cardiac function. However, only a limited number of studies investigated whether AT infiltration of skeletal muscles is related to cardiac function as presented in the included studies in this review [23–25]. Another review article has also discussed the possibility of IMAT to influence cardiovascular system [35]. Using AT infiltration may be advantageous in relation to cardiac function because quantification of AT infiltration provides an estimation of fat volume in vital organs, such as skeletal muscles [29,30,40,41], which could be more accurate than overall body composition assessment. Furthermore, overall body composition is often considered when BMI is increased, leaving those who may have low BMI and increased AT infiltration undetected for cardiac risks. However, there is limited evidence on the usability of AT infiltration as a biomarker for cardiac function, warranting further research.

Evidence shows that insulin sensitivity plays a key role in AT infiltration [29,30,40,41] and cardiac myopathy [34], thereby influencing the relationship between these two factors [29,40]. In addition, BMI may contribute to the relationship between AT infiltration and cardiac function. The two included cross-sectional studies [23,24] recruited participants who were overweight (BMI from 25 to 30), which may have contributed to the association between AT infiltration and cardiac function. To support this observation, the third included longitudinal study [25], classified participants into tertiles based on the IMAT level. The high IMAT group showed higher heart failure events, and high BMI levels. Thus, the association between AT infiltration and cardiac function may be explained by insulin sensitivity and BMI level.

While IMAT and IntraMAT differ biologically in formation [29,31] with IMAT being modifiable and can be avoided [42,43], the results of this review showed that higher concentration of either IMAT or IntraMAT was associated with impaired cardiac function and exercise capacity, independent of general obesity. Moderate intensity aerobic exercise, either alone or combined with resistance training, may help in reducing IMAT in skeletal muscles [44]. However, it is unclear whether reducing AT infiltration of skeletal muscles would improve cardiac function or decrease heart failure events. Our findings may instigate future studies to explore the effects of exercise on reducing AT infiltration and improving cardiac function.

Our study does not preclude limitations. For instance, the included studies in this review used observational study designs [23–25], leaving uncertainty about the causal relationship between AT infiltration and cardiac function. However, all studies achieved good quality based on aspects of internal validity measured by SQAT, providing confidence in the findings of these studies. While all included studies used observational designs, the goal of this scoping review was not to infer causality, but to summarize and map existing evidence on the association between AT infiltration and cardiac function in human populations. However, the consistency of our findings across diverse populations supports the clinical signal that warrants further investigation through mechanistic and interventional studies. The lack of randomized controlled trial studies in the included literature highlights a significant gap in the field that future research should address.

This review provides several clinical implications. First, assessment of AT infiltration of skeletal muscles poses clinical significance for cardiac function in addition to its use in assessing the risk of frailty and muscle quality. Additionally, increased AT infiltration may be associated with risks to cardiac function, considering demographic and anthropometric factors such as age and BMI. For instance, individuals with low BMI may still be at risk of cardiac impairment and have increased AT infiltration of the skeletal muscle. AT infiltration may explain cardiac risk even in individuals with low BMI, thus separating its effect from general adiposity. This is important because increased fat is often considered only when BMI reflects overweight or obesity. Finally, this review summarizes the literature on the identification of a new biomarker of cardiac function associated with advanced age.

## Conclusions

The findings of this review denoted limited, good-quality evidence that increased AT infiltration of skeletal muscles, including IMAT and IntraMAT, is associated with impaired cardiac function parameters. These findings highlight that skeletal muscle AT infiltration, distinct from general obesity, may represent a novel biomarker for cardiac risk stratification. AT infiltration provides further insight into fat distribution, despite obesity level. Future studies may examine if decreasing AT infiltration of the skeletal muscle would enhance cardiac function in populations at risks of increased AT infiltration (e.g. older adults).

## Supplementary Material

Supplement 1.docx

## Data Availability

Data sharing is not applicable to this article as no new data were created or analysed in this study. This is a review study and data synthesis in this review included information from the retrieved articles. The data used were obtained directly from the finally included articles which have been previously published articles.
