# Supplementary material for: Association of adipose tissue infiltration with cardiac function: scoping review
Source: Adipocyte. 2025 Apr 10;14(1):2489467. doi: 10.1080/21623945.2025.2489467 (PMC11988230; doi:10.1080/21623945.2025.2489467)
Supplement: Supplement 1.docx [file KADI_A_2489467_SM1974.docx]

Supplement 1. Detailed search strategy with search terms and database queries.

| **No.** | **Database** | **Search Terms** | **Records Retrieved** |
| --- | --- | --- | --- |
| 1 | PubMed | (("intermuscular fat"[Title/Abstract] OR "intermuscular fat"[Other Term] OR "intramuscular fat"[Title/Abstract] OR "intramuscular fat"[Other Term] OR "Adipose tissue*"[Title/Abstract] OR "Adipose tissue"[Other Term] OR "fat*"[Title/Abstract] OR "fat"[Other Term] OR "body mass index"[Title/Abstract])) AND (("systolic function"[Title/Abstract] OR "ejection fraction"[Title/Abstract] OR "cardiac output"[Title/Abstract] OR "Stroke volume"[Title/Abstract] OR "cardiac index"[Title/Abstract] OR "swan-ganz catheter"[Title/Abstract] OR "echocardiograph"[Title/Abstract] OR "Stress echo"[Title/Abstract] OR "ejection acceleration time"[Title/Abstract] OR "global longitudinal strain"[Title/Abstract] OR "fractional shortening"[Title/Abstract] OR "fractional area change"[Title/Abstract] OR "left ventricle ejection fraction"[Title/Abstract])) NOT ((“animal studies”[Title/Abstract] OR “animal study”[Title/Abstract] OR “rat*” [Title/Abstract] OR “heart failure”[Title/Abstract] OR “sarcopenia”[Title/Abstract] OR “knockout*”[Title/Abstract] OR “Review”[Title/Abstract])) | 2757 |
| 2 | CINAHL | (MH "intamuscular fat" OR TI ( intermuscular-fat OR intamuscular fat OR Adipose-tissue OR fat OR body-mass-index ) OR AB ( intermuscular-fat OR intamuscular fat OR Adipose-tissue OR fat OR body-mass-index )) AND (TI ( systolic-function OR ejection-fraction OR cardiac-output OR Stroke-volume OR cardiac-index OR swan-ganz-catheter OR echocardiograph OR Stress-echo OR ejection-acceleration-time OR global-longitudinal-strain OR fractional-shortening OR fractional-area-change OR left-ventricle-ejection-fraction ) OR AB ( systolic-function OR ejection-fraction OR cardiac-output OR Stroke-volume OR cardiac-index OR swan-ganz-catheter OR echocardiograph OR Stress-echo OR ejection-acceleration-time OR global-longitudinal-strain OR fractional-shortening OR fractional-area-change OR left-ventricle-ejection-fraction ) OR MH “ejection-fraction”) NOT (MH "animal-studies" OR MH "review" OR TI ( animal-studies OR rat* OR review OR sarcopenia OR hear-failure* OR knockout* ) OR AB ( animal-studies OR rat* OR review OR sarcopenia OR hear-failure* OR knockout* )) | 544 |
| 3 | Scopus | TITLE-ABS-KEY ( intermuscular-fat OR intamuscular fat OR Adipose-tissue OR fat OR body-mass-index ) AND TITLE-ABS-KEY ( systolic-function OR ejection-fraction OR cardiac-output OR Stroke-volume OR cardiac-index OR swan-ganz-catheter OR echocardiograph OR Stress-echo OR ejection-acceleration-time OR global-longitudinal-strain OR fractional-shortening OR fractional-area-change OR left-ventricle-ejection-fraction ) NOT TITLE-ABS-KEY ( animal-studies OR rat* OR review OR sarcopenia OR hear-failure* OR knockout*) | 3 |
